# Gene expression-based dissection of inter-histotypes, intra-histotype and intra-tumor heterogeneity in pediatric tumors

**DOI:** 10.1038/s41598-022-20536-6

**Published:** 2022-10-25

**Authors:** Sara Ciceri, Andrea Carenzo, Maria Federica Iannó, Alessia Bertolotti, Carlo Morosi, Roberto Luksch, Filippo Spreafico, Paola Collini, Paolo Radice, Maura Massimino, Loris De Cecco, Daniela Perotti

**Affiliations:** 1 Molecular Bases of Genetic Risk and Genetic Testing Unit, Department of Research, Fondazione IRCCS Istituto Nazionale Dei Tumori, Via Venezian 1, 20133 Milano, Italy; 2 Molecular Mechanisms Unit, Department of Research, Fondazione IRCCS Istituto Nazionale Dei Tumori, Via Venezian 1, 20133 Milano, Italy; 3 Integrated Biology Platform, Department of Applied Research and Technology Development, Fondazione IRCCS Istituto Nazionale Dei Tumori, Milan, Italy; 4 Department of Pathology and Laboratory Medicine, Fondazione IRCCS Istituto Nazionale Dei Tumori, Milan, Italy; 5 Department of Radiology, Fondazione IRCCS Istituto Nazionale Dei Tumori, Milan, Italy; 6 Pediatric Oncology Unit, Department of Medical Oncology and Hematology, Fondazione IRCCS Istituto Nazionale Dei Tumori, Milan, Italy

**Keywords:** Cancer, Embryonal neoplasms, Paediatric cancer

## Abstract

Intra-tumor heterogeneity (ITH) fosters tumor evolution, resistance to therapy, and relapse. Recently, many evidence have been accumulated on the occurrence of genetic ITH in pediatric cancers. With this study we aimed to address the downstream effects that genetic and epigenetic ITH, and tumor-microenvironment interactions may produce within a tumor mass. To this aim, we investigated by high-throughput gene expression multiple samples of 5 hepatoblastomas, 5 neuroblastomas, 5 rhabdomyosarcomas, and 5 Wilms tumors. Principal component analysis, single sample hallmark gene sets analysis, and weighted gene co-expression network analysis were performed on gene expression data. We observed that the different tumors clustered by histotype, and then by case, and in addition, a variable degree of ITH was visible in all the investigated cases. The ITH highlighted in this study can represent a challenge in tumor treatment since we demonstrated that different druggable hallmarks and targets may be heterogeneously present within the same tumor mass, and this can potentially lead to therapeutic failure. Despite this heterogeneity, we could highlight some commonalities among the different histotypes investigated, supporting the feasibility to move in the clinic from a histotype-driven to a target-driven, sometimes agnostic, approach at least in some cases.

## Introduction

The occurrence of intra-tumor heterogeneity (ITH) is well known by pathologists, who have long been describing the different morphological features that cells within a single tumor mass may exhibit^1^. Even radiologists are aware of this phenomenon, since tumors can display a radiologically inhomogeneous appearance^2–4^. Despite of this, at present, diagnosis and therapy stratification of patients are in most cases based on a single tumor bioptic specimen, assuming that the sampled fragment can adequately represent the entire tumor mass. This assumption is now being questioned, thank to the improvements of molecular technologies and the availability of advanced approaches to dissect and report ITH.

ITH is not surprising considering that cancer is a dynamic disease. In fact, cancer evolves in time and space, accumulating genetic events that occur stochastically in dividing cells and give rise to distinct clones from the initial cancer cell(s). Through this process, clonal cell populations progressively become more genetically distant, as the tumor grows and further mutations accumulate randomly (reviewed in^5–9^). Another driver of ITH is the phenotypic plasticity, with different tumor cells resembling the hierarchical organization occurring in normal tissue, in which stem-like cells can give rise to a progeny of more differentiated cells. Cancer stem cells are normally not genetically distinct from their more differentiated descendant cells, but they are usually more quiescent and resistant to treatments. They express transcriptional programs consistent with those of normal stem cells, and present a more promiscuous chromatin organization, which allow them this plasticity. Therefore, also the differences in DNA methylation, histone modifications and chromatine accessibility, and thus in the global epigenome of the distinct tumor cell populations, contributes to increase ITH (reviewed in^5–9^). ITH provides genetically and epigenetically different cellular clones upon which selection and Darwinian evolution can act. Extrinsic factors further modulating ITH are due to the effect of the tumor microenvironment which comprises a set of variables including blood vessel density, extracellular matrix composition and stiffness, and immune system cells. In fact, all these microenvironment factors affect all the different tumor cell clones (reviewed in^5–9^).

Genetically acquired ITH has been demonstrated in a variety of human tumors, including different pediatric malignancies^10–18^. In particular, the analysis of the prevalence of genetically distinct clones in 54 childhood tumors, including neuroblastomas (NBs), rhabdomyosarcomas (RMSs) and Wilms tumors (WTs), disclosed that the cells of primary tumors can progress to up to four distinct evolutionary trajectories in different areas^10^.

With this work we aimed to dissect, through gene expression analysis, the phenomenon of ITH within four pediatric tumor histotypes, using multiple tissue samples for each case in 5 hepatoblastomas (HBs), 5 NBs, 5 RMSs and 5 WTs. Although we recognize that our results represent very preliminary data, obtained in a specific set of cases, we are confident that some suggestions may be drawn.

## Materials and methods

### Patients and samples

Based on the availability of complete clinical, radiological and pathological information, as well as of multiple formalin-fixed, paraffin embedded (FFPE) tumor tissue blocks (FFPE block = sample) from spatially distinct areas of the primary tumor mass (tumor mass = case), possibly with different morphologies, 5 HBs, 5 NBs, 5 RMSs, and 5 WTs were selected. RMSs were represented by alveolar, embryonal, and sclerosing types. Four different FFPE blocks were selected for all patients with the exception of one NB case, in which only 3 different blocks were available, for a total of 79 samples. The percentage of viable tumor in each FFPE block was assessed by an expert pediatric pathologist and macrodissection was performed when necessary. Clinical, radiological and pathological data of the selected patients, together with the microscopical description of the single samples have been collected and reported in detail in Supplementary Table 1. All research was performed in accordance with relevant guidelines/regulations.

### RNA Extraction from FFPE

Total RNA was isolated from the 79 samples using miRNeasy FFPE Kit (Qiagen, Valencia, CA, USA), and the procedure was automated on a QIAcube Robotic workstation. The RNA extracted was quantified using Qubit™ RNA HS Assay Kit on a Qubit fluorometer (ThermoFisher, Waltham, MA, USA) while RNA integrity was assessed using Agilent RNA ScreenTape on a 4200 TapeStation, (Agilent Technologies, Santa Clara, CA, USA).

### Gene expression profiling

Probe synthesis was performed using the Clariom S Pico Assay starting from total RNA. Total RNA was reverse transcribed, amplified, fragmented, biotin-labeled, and then hybridized to the Affymetrix GeneChip Human Clariom S Array (ThermoFisher Scientific, Waltham, MA, USA). Washing and staining procedures were performed using the GeneChip Hybridization, Wash and Stain Kit (ThermoFisher Scientific) on a GeneChipHybridization Oven 645 (ThermoFisher Scientific) according to the manufacturer’s protocol. Scanning was performed on Affymetrix GeneChip Scanner 3000 TG System and primary data were collected using GeneChip™ Command Console™ (AGCC) Software (ThermoFisher Scientific)^19^. Quality controls have been implemented into the experimental workflow according to the manufacturer’s protocol to check RNA quality, probe synthesis, and hybridization performance. Following manufacture’s guidelines, quality check was performed by analyzing the area under the curve (AUC) of the probe set summary ability to distinguish between positive and negative controls (e.g., exon from intron sequences). Our dataset reaches mean AUC = 0.93 (range 0.88–0.96) confirming the good quality of the profiling.

### Data availability

All microarray data were compliant to MIAME (Minimum Information About a Microarray Experiment) guidelines and were deposited into the Gene Expression Omnibus (GEO) database of NCBI (National Center for Biotechnology Expression) (http://www.ncbi.nlm.nih.gov/geo/), with accession numbers GSE197147.

### Bioinfomatic analyses

All bioinformatic analyses were performed using the R statistical software^20^ version 4.0.5 and Bioconductor (version 3.12) packages^21^. For visualization purposes ggplot2 R package (version 3.3.6) was used.

Data normalization was performed using the rma function of the oligo R package^22^ with default parameters and averaging the expression values of probes coding the same gene. The false discovery rate (FDR)^23^ was used to correct *p*-value for multiple testing and we imposed FDR < 0.05.

Chromosomal instability (CIN) was assessed following the CIN70 developed by a bioinformatic approach to identify genes associated with total functional aneuploidy across multiple types of cancer^24^. A score for CIN70 was computed as ssGSEA method avaible in the R package GSVA^25^.

The tumor mutation burden (TMB) was calculated by a LASSO regression model (“19-TMB-related-gene model”) developed across multiple cancer types^26^.

Somatic copy number variations (CNVs) were imputed by using the opoSOM v2.15 R package^27^ that offers enrichment analyses for genes sets relating to chromosomal positions and plotting.

### Principal Component Analysis (PCA)

We applied a mathematical, simple, and intuitive way to represent the tumor heterogeneity in our cohort through gene expression data and we used a principal component analysis (PCA) approach. A common method for determining the number of principal components (PCs) to be retained is a graphical representation known as a scree plot and selecting the components before the “elbow” in the curve; as a matter of fact, after that point, the line flattens out and an any additional PCs would add relatively little information. By looking for the presence of an elbow in our scree plot, the first 6 principal components were retained reaching a total explained variance = 48.77% (Supplementary Fig. 1). Afterwards, we used those PCs to compute the euclidean distance between each pair of samples. Moreover, for visualization purposes, we computed two types of centroids: (i) four centroids corresponding to the center of a histotype (average position of samples with the same histotype); (ii) twenty centroids corresponding to the center of a tumor (average position of samples coming from the same tumor). After computing the euclidean distance between each sample and the resulting 24 centroids, we represented the heterogeneity of our cohort at the intra-histotype and intra-tumor level by means of four simple summary metrics (Supplementary Table 2):

- Mean pairwise distance = *mean*_*i,j*_* d(i, j)*, the average distance between each two samples *i* and *j* corresponding to the same histotype or tumor;
- Maximum pairwise distance = *max*_*i,j*_* d(i, j)*, the maximum distance between each two samples *i* and *j* corresponding to the same histotype or tumor;
- Mean centroid distance = *mean*_*i*_* d(i, c)*, the average distance between a sample *i* and its corresponding histotype or tumor centroid *c*;
- Max centroid distance = *max*_*i*_* d(i, c)*, the maximum distance between a sample *i* and its corresponding histotype or tumor centroid *c*;

where *d(i, j)* and *d(i, c)* represent the euclidean distance between the six-dimensional representation of sample *i* and sample *j* and between sample *i* and a centroid *c*, respectively. Our selection of matrix distance was determined by the nature of the data since our microarray data were normalized. Despite the fact that there exist many different ways to compute a distance measure, we opted for simplicity and intuitiveness in order plot data in a 2-D space. In addition, we considered the two kinds of mean euclidean distance as a general measure of heterogeneity and the two kinds of maximum distance as a way to penalize more heterogeneous tumors or histotypes.

### Single sample z-score analysis

Single sample Hallmark Gene Set (HGS) analysis was performed on the four histotypes separately after standardizing values so that a HGS more or less enriched in one histotype was comparable with the same HGS in the other three histotypes. In order to investigate the enrichment of the HGS in each tumor sample, we used the z-score method implemented in the R package GSVA^25^. Expression values of genes composing the sets were centered by their mean and scaled by their standard deviation (z-scores). Then, the single sample z-score was computed as the sum of normalized expression values divided by the square root of the gene set size. HGS resulted more or less enriched depending on the single sample z-score value above or below zero, respectively.

Moreover, in order to consider as expressed the druggable genes of the HGS, we considered a log2 expression of 6 because this was the median of log2 expression for all genes in all samples.

### Weighted gene co-expression network analysis (WGCNA)

Bioinformatics methods allow correlations between gene expression patterns in different samples to be described, as well as the correlation of gene expression patterns among individual samples, and this approach is commonly used in WGCNA^28^. This method simplifies the process of complex data processing to assist in the study of gene modules most likely to be involved in biological functions. The module eigengene (ME) is a representative value of each module, which is equivalent to the first principal component and explains the largest proportion of variance of the module genes. Power = 12, mergeCutHeight = 0.25 and a maxBlockSize = 20,000 were chosen as parameters in the function blockwiseConsensusModules.

### Over-representation analysis

Nineteen consensus modules were found with WGCNA and over-representation analyses were applied through the R package gprofiler2^29^. The following gene set collections were explored for over-representation in consensus modules: Gene Ontology, KEGG, Reactome, WikiPathways, regulatory targets from miRTarBase and TRANSFAC, Human Protein Atlas and Human Phenome Ontology.

### Ethical approval

The study was approved by the Ethical Committee of the Fondazione IRCCS Istituto Nazionale dei Tumori, Milan, Italy, (protocol: INT 112/18; 27 june 2018). Informed consent was obtained from the parents or the guardians of all patients.

## Results

**Figure 1:**
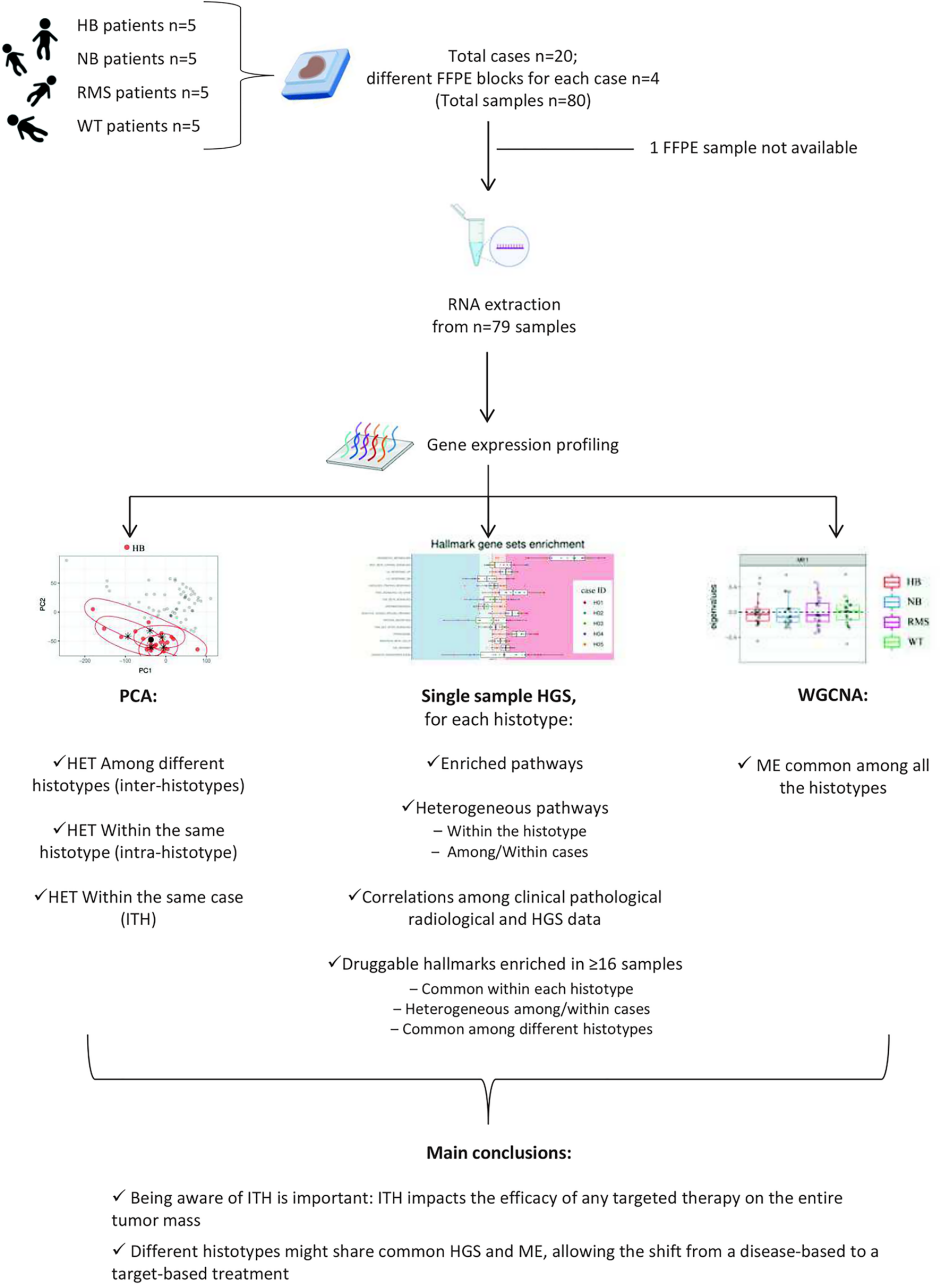
Flowchart. Flowchart of the present work representing material, methods, obtained results and conclusions. WT: Wilms Tumor; NB: neuroblastoma; HB: hepatoblastoma; RMS: rhabdomyosarcoma; n: number; FFPE: formalin-fixed, paraffin-embedded; PCA: Principal Component Analysis; HGS: Hallmark Gene Sets; WGCNA: Weighted gene co-expression network analysis; HET: heterogeneity; ITH: intra-tumor heterogeneity; ME: module eigengenes. The figure was created with with https://BioRender.com.

The flowchart of the present work is depicted in Fig. 1.

### Inter-histotypes heterogeneity, intra-histotype heterogeneity, and intra-tumor heterogeneity (ITH)

**Figure 2:**
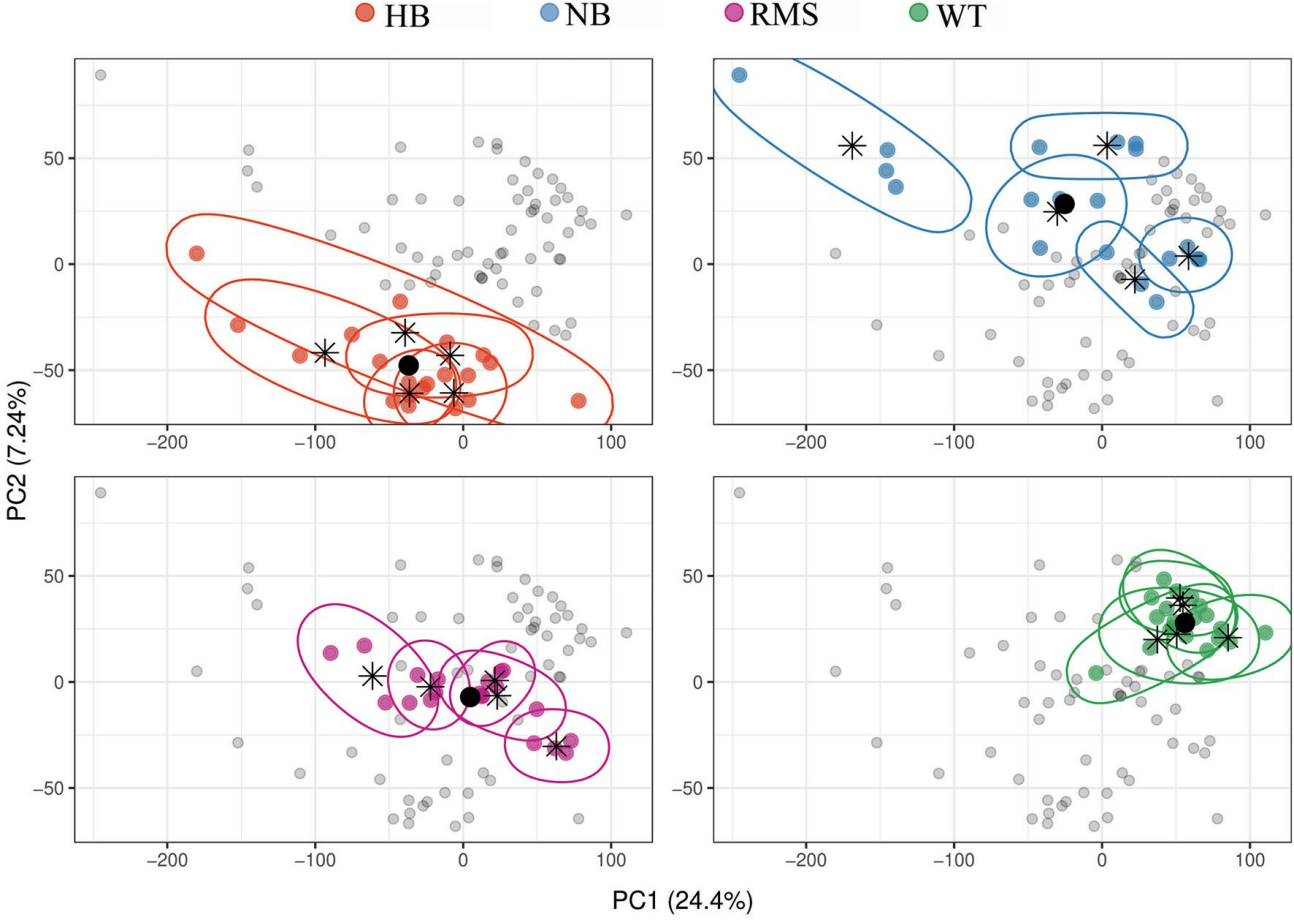
Principal Component Analysis. The plot shows a Principal Component Analysis (PCA) made on the gene expression profiles of 79 samples (dots). The x- and y-axis represent the first two principal components, PC1 and PC2 respectively, which together explain about 31.6% of the total variance. In each panel samples for each histotype are highlighted by different colors (HB: red dots; NB: blue dots; RMS: purple dots; WT: green dots). Samples coming from the same case are encircled and stars represent their center, whereas big black dots represent the center of the histotype. Grey dots represent samples of the other histotypes.

The described principal component analysis (PCA**)** approach applied on the different samples belonging to the four different pediatric histotypes allowed the clustering of the samples according to the histotypes (Fig. 2). In particular, Wilms tumor (WT) seemed the most homogeneous histotype, whereas neuroblastoma (NB) looked as the most heterogeneous, with rhabdomyosarcoma (RMS) and hepatoblastoma (HB) being “mildly” heterogeneous histotypes. Within each histotype, each case tended to cluster from the others (intra-histotype heterogeneity), despite of this, a variable degree of ITH was appreciable as shown in Supplementary Table 3, reporting both intra-histotype and intra-case distances.

We also explored the presence of main genomic alterations able to depict inter-histotypes, intra-histotype and intra-tumor heterogeneity. We confirmed the low impact of mutational load or structural copy number variation in pediatric cancers. As a matter of fact, tumor mutation burden (TMB) has a low range among samples and no clear separation was found among histotypes by interpolation of TMB and CIN70 signatures (Supplementary Fig. 2). Similarly, no significant copy number alterations were found when gene enrichment was related to chromosal gene position (Supplementary Fig. 3).

### Single sample Hallmark Gene Set (HGS) in the four investigated histotypes

Complete HGS single sample z-scores are reported in Supplementary Table 4 and depicted in Supplementary Figs. 4, 5, 6, 7.

According to our PCA analysis, the HB histotype resulted to be “mildly” heterogeneous. Excluding HGS that were organ-specific, the most enriched were TNFA_signaling_via_NFKB, Apoptosis and Complement. The highest variability among HB samples was observed in TNFA_signaling_via_NFKB, Epithelial_mesenchimal_transition, MYC_targets_v1, MTORC1_signaling Oxidative_phosphorilation and the immune HGS (Interferon_gamma_response, Interferon_alpha_response, Inflammatory_response, Complement). A minor variability was seen in the least enriched HGS: Mitotic_spindle, G2M_checkpoint, E2F_targets, DNA repair (Supplementary Table 4 and Supplementary Fig. 4).

Among the four histotypes analyzed, NB was the most heterogeneous. Apical_surface, and Hedgehog_signaling were the HGS most enriched in NB samples. The most heterogeneous HGS were Epithelial_mesenchimal_transition, TNFA_signaling_via_NFKB, the immune HGS (Interferon_gamma_response, Interferon_alpha_response, Inflammatory_response, Complement), and Oxidative_phosphorilation. The WNT_BETA_catenin_signaling pathway involved in the development of NB was poorly enriched in our cases (Supplementary Table 4 and Supplementary Fig. 5).

RMS resulted to be a “mildly” heterogeneous histotype according to our PCA. HGS enriched in RMS samples were MYC_targets_v1, Hypoxia, and TP53_pathway. The most variability among RMS samples was observed in Myogenesis, Oxidative_phosphorilation, TNFA_signaling_via_NFKB, G2M_checkpoint, E2F_target, and Epithelial_mesenchymal_transition HGS. A minor variability was seen in TP53_pathway, NOTCH_signaling, IL6_JACK_STAT3_signaling HGS (Supplementary Table 4 and Supplementary Fig. 6).

As already shown by PCA, the least variability among samples of WT cases was also evident in single sample HGS analysis; in general, the different samples appeared very close to each other indicating very similar gene expression levels. Among the biological processes more enriched in WT cases there were the proliferative HGS (G2M_checkpoint, E2F_targets, MYC_targets_v1, Mitotic _spindle). Epithelial_mesenchimal_transition, MYC_targets_v1, G2M_checkpoint, and E2F_targets were the most heterogeneous HGS. The immune HGS were poorly enriched in our WT cases (Supplementary Table 4 and Supplementary Fig. 7).

### Intra-histotype heterogeneity and ITH in single sample HGS enrichment in the four histotypes investigated

A various degree of intra-histotype heterogeneity and ITH was observed in all the different histotypes analyzed (Supplementary Table 4 and Supplementary Figs. 4, 5, 6, 7).

In particular, considering the most enriched and/or more heterogeneous HGS, in HB cases, TNFA_signaling_via_NFKB, and Complement were enriched in all samples of cases 1, 2, 3, 4 but only in 1/4 or 2/4 samples of case 5, respectively; Epithelial_mesenchimal_transition, was enriched in all samples in case 2 but only in 2/4 in cases 1 and 5, and in 1/4 in cases 3 and 4; MTORC1_signaling was enriched in all samples in cases 1 and 3 but only in 3/4 samples in case 2 and in 2/4 samples in cases 4 and 5; Interferon_alpha_response was enriched in all samples in cases 1 and 3, in 3/4 of case 2 and in 2/4 of cases 4 and 5; Interferon_gamma_response was enriched in all samples in cases 1, 2 and 3, and in 2/4 in cases 4 and 5; Inflammatory_response was enriched in all samples in cases 2, 3, 4 in 3/4 samples of case 1, and in 1/4 of case 5; Oxidative_phosphorilation was enriched in all samples of cases 1 and 3, in 3/4 of case 2 and in 1/4 of cases 4 and 5 (Supplementary Table 4 and Supplementary Fig. 4).

In NB cases, TNFA_signaling_via_NFKB was enriched in all samples in cases 2 and 5, was enriched in 1/4 samples in case 1, and poorly enriched in all samples of cases 3 and 4; Epithelial_mesenchimal_transition was enriched in all samples in cases 2 and 5, and poorly enriched in cases 1, 3 and 4; Interferon_gamma_response, Interferon_alpha_response, Inflammatory_response, as well as Complement, were enriched in all samples of cases 2 and 5, in one sample of case 1, and poorly enriched in all samples of cases 3 and 4; Oxidative_phosphorilation was enriched in all samples in cases 2 and 5, poorly enriched in all the samples of the remaining cases (Supplementary Table 4 and Supplementary Fig. 5).

In RMS cases, TNFA_signaling_via_NFKB was enriched in all samples of cases 2, 3, in 3/4 samples of patient 4, and poorly enriched in cases 1 and 5; G2M_checkpoint and E2F_targets were enriched in all samples of cases 1, 3 and 4, poorly enriched in all samples of cases 2, 5; Epithelial_mesenchimal_transition was enriched in all samples of cases 1, 2, 3 and 4 but only in 1/4 of case 5; Oxidative_phosphorylation was enriched in all samples of cases 2 and 4, in 2/4 samples of case 3, poorly enriched in cases 1 and 5; Myogenesis was enriched in all samples of cases 1, 2, 4, 5 and poorly enriched in case 3 (Supplementary Table 4 and Supplementary Fig. 6).

In WT cases, the MYC_targets_v1 and E2F_targets were enriched in all samples of all cases but case 3, in which only 2/4 samples were enriched for these HGS; the G2M_checkpoint was similarly enriched in all samples of all cases but case 3 in which only 1/4 sample was enriched. The Epithelial_mesenchimal_transition was enriched in all the investigated samples in cases 3 and 5, in 2/4 samples investigated in case 4, and poorly enriched in cases 1 and 2 (Supplementary Table 4 and Supplementary Fig. 7).

### Correlations among clinical-radiological-histopathological and HGS data

We investigated possible correlations among clinical, radiological, histopathological and HGS data in the different cases investigated (Supplementary Tables 1 and 4). Representative radiological and histological images of some of the reported cases are shown in Supplementary Figs. 8, 9, 10.

In HB, the radiological picture was homogeneous in cases 2 and 3, all samples of cases 2 and 3 were consistently histologically homogeneous (all distinct samples of the case were constituted by the same type of cells). Remaining cases were heterogeneous both radiologically and histologically. Patient 1 had progressive disease and died, but we could not find any difference in HGS enrichment with the remaining cases (Supplementary Tables 1 and 4).

All NB cases resulted homogeneous from a radiological view, although samples of cases 1, 2 and 3 were histologically heterogeneous. The two cases (2 and 5) with metastatic disease at diagnosis displayed a higher number of HGS enriched compared to the other cases, such as Epithelial_mesenchimal_transition, Interferon_alpha_response, Interferon_gamma_response, Inflammatory_response, Oxidative_phosphorilation, Apoptosis, Hyopoxia, KRAS_signaling_up, TGF_beta_signaling, TNFA_signaling_via_NFKB. The two cases (2 and 5) were among the three NB cases treated for high risk disease (2, 4 and 5), and one out of these two cases (case 2) relapsed and died of disease (Supplementary Tables 1 and 4).

All RMS cases were radiologically heterogeneous, however, only case 3 displayed histologically heterogeneous samples. Cases 1, 3 and 4 had a relapsing disease, and intriguingly, they showed MYC_target_v2, E2F_targets, G2M_checkpoint HGS enrichment compared to the remaining cases (Supplementary Tables 1 and 4).

In WT cases, we observed that although the radiological presentation could be heterogeneous (cases 2, 3), from the histological point of view all the samples of case 2 were homogeneous. Cases 4 and 5 had a prevalence of stromal component, and consistently in these cases the WNT_beta_catenin_signaling pathway was enriched; case 2 relapsed, and E2F_targets, G2M_checkpoint, MYC_target_v1, and Mitotic_spindle were very enriched in this case (Supplementary Tables 1 and 4).

### Druggable pathways among the different histotypes investigated

Considering the different histotypes investigated, on an exploratory basis, we selected, starting from the HGS, a number of druggable pathways (Epithelial_mesenchimal_transition, Oxidative_phosphorilation, Apoptosis, Hypoxia, E2F_targets, G2M_checkpoint, MYC_targets_v1, MYC_targets_v2, KRAS_signaling, MTORC1_signaling, Hedgehog_signaling and WNT_BETA_catenin_signaling) to find the most commonly enriched for each histotype, and then to explore the possibility of the existence of common druggable pathways among the different histotypes. Each histotype was separately examined, and for each one, among the above listed druggable pathways, only those in which the HGS was enriched in least 16/19–20 samples were further considered. For the most commonly enriched pathway of each histotype, we selected a number of druggable molecules to assess their expression in the different samples (Supplementary Table 5).

In HB, the Apoptosis and the MYC_target_v2 HGS were enriched in 16/20 samples, the Hypoxia HGS was enriched in 19/20 samples. Exploiting Hypoxia, the druggable target PRKDC was expressed in all the samples, whereas CHEK1 was heterogeneously expressed both among cases and within the same case, and PARP1 was not expressed.

In NB, the Hedgehog_signaling HGS was enriched in 16/19 samples, and the druggable targets GLI1 and GLI2 were expressed in all the samples whereas SMO was expressed in all but one sample.

In RMS, Hypoxia and MYC_target_v1 HGS were both enriched in 16/20 samples, the Epithelial_Mesenchymal_Transition HGS was enriched in 17/20 samples; exploiting this last one, the druggable target AXL was expressed in all samples, whereas TGFB1 was heterogeneously expressed both among cases and within the same case.

In WT, the E2F_target HGS was enriched in 18/20 samples, the G2M_checkpoint HGS was enriched in 17/20 samples, and the MYC_target_v1 HGS was enriched in 18/20 samples. Considering the E2F_target HGS in WT, the druggable genes CDK4 and CDK6 were expressed in 20/20 samples, whereas NEK2, SGO1, and TTK resulted heterogeneously expressed both among cases and within the same case.

As above described, this analysis also highlighted possible druggable pathways in common among different histotypes, in fact, MYC_target_v1 was enriched both in WT and RMS, and Hypoxia both in RMS and HB, showing the possibility of target-driven therapeutical strategies common among different histotypes.

### Weighted gene co-expression network analysis (WGCNA)

**Figure 3:**
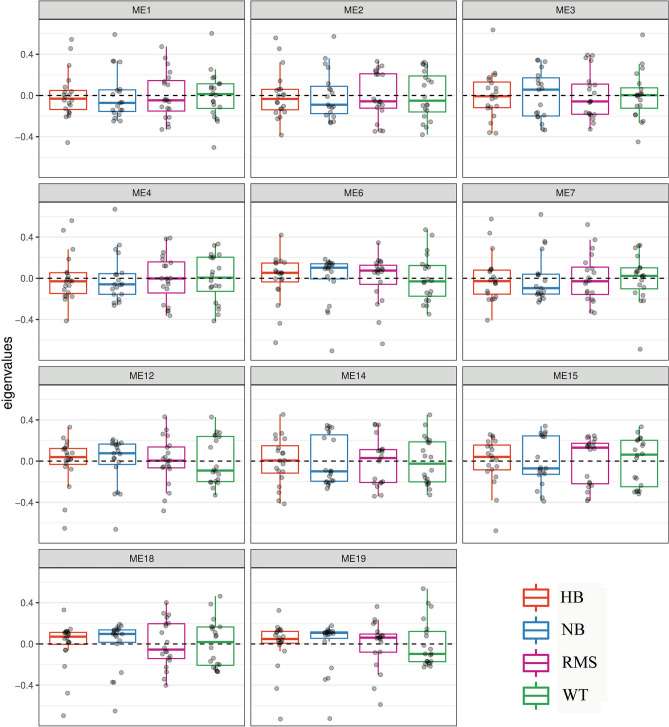
Weighted Gene Co-expression Network Analysis (WGCNA) Module Eigengenes (ME). The boxplots show ME scores (y-axis) across histotypes (x-axis) for modules obtained through a WGCNA. The genes included in all ME and the top functions are reported in Supplementary Table 6. HB: red; NB: blue; RMS: purple; WT: green.

To define the expression relationships among the four histotypes investigated, a consensus module analysis approach with WGCNA was applied. The procedure includes a multi-step processing: i) the correlation coefficients between any two genes was computed, and the relationship among genes was inferred by the weighted value of the correlation coefficient in the network to define scale-free networks; ii) a hierarchical clustering tree was constructed from the correlation coefficients between genes; iii) highly correlated gene modules were merged together and 19 module eigengene (ME) were obtained. Figure 3 shows the ME among the different cases of the four histotypes highlighting pathway/biological functions. The genes included in each ME and the top functions are reported in Supplementary Table 6.

Among the identified ME, ME1 involves mRNA metabolic and catabolic processes, ribosome, and translational processes, cellular macromolecule catabolic process and ribonucleoprotein complex; ME3 protein targeting to membrane/endoplasmic reticulum, ribosome, translation, mRNA catabolic processes; ME6 cell cycle and mismatch repair; ME14 and ME15 immune system; ME18 mRNA splicing and processing; ME19 intracellular transport and cellular localization. Remaining ME are described in detail in Supplementary Table 6.

## Discussion

Nowadays, precision medicine is entering the clinics also for childhood tumors, in which biomarkers are being used for diagnosis and risk stratification, and the technical improvements are making our knowledge on the molecular landscapes of these diseases more and more detailed. This has led to realizing that, if we ground our analyses on the examination of a single sample of the whole tumor mass, as it has usually been done until now, we could have an incomplete picture of the disease. Genetic ITH has been widely reported in many adult tumors. These tumors typically develop over long periods of time before being diagnosed, and often display branched evolutionary trajectories^30–33^. In contrast, pediatric tumors evolve over shorter period of time and have a lower mutational burden. Thus they could be expected to have less complex evolutionary histories^34,35^ and until recently investigations on their level of genetic ITH were almost missing.

Genetic ITH has been now demonstrated in different pediatric tumors^10–18^, and with our work we sought to contribute to add evidence to this phenomenon, assessing the extent of intra-tumor gene expression heterogeneity in some selected pediatric tumors at onset. To this aim, we performed gene expression profiling of 4 spatially distinct tumor areas in patients affected by HB, NB, RMS and WT (5 cases for each histotype, for a total of 79 samples). As expected, PCA disclosed differences among the different histotypes, which clustered separately one from the other, and showed a variable degree of intra-histotype heterogeneity, being NB and WT the most and the less heterogeneous histotypes, respectively. Within each histotype, each case also clustered separately from the others, and intriguingly, also within each case, a certain degree of ITH was always evident among the different samples investigated. The same scenario was reproduced by single sample HGS analysis. The inter-histotypes heterogeneity was evident from the different HGS which were enriched in the different histotypes. Likewise, intra-histotype and ITH was strikingly evident from single sample HGS data: a variable level of heterogeneity was visible in all the studied histotypes for all the HGS, but this heterogeneity became striking when considering the most enriched and/or more heterogeneous HGS. In fact in this analysis some of the investigated cases/samples were observed within the more enriched and others the poorly enriched ranges.

Then we investigated, on exploratory level, a selected number of HGS in which druggable targets are present. In each histotype separately, we considered a HGS as commonly enriched when enriched in at least 16/19–20 samples. In our cases, commonly enriched HGS in HB were Apoptosis, MYC_target_v2, and Hypoxia; in NB the Hedgehog_signaling HGS; in RMS the Hypoxia, MYC_target_v1 and Epithelial_Mesenchymal_Transition; in WT the E2F_target, the G2M_checkpoint, and the MYC_target_v1. We found that although by HGS a druggable pathway might look enriched in a defined histotype, the different druggable molecules belonging to that pathway could be differently expressed among the different individual cases within that histotype, but even more intriguingly, they could be differently expressed within the same tumor mass. The implications of these observations are important: a patient treated with a drug for a target which is heterogeneously expressed within the tumor mass, will have in fact tumor cells not affected by the therapy.

Our analysis showed furthermore the presence of HGS in which druggable targets are present, commonly enriched among different histotypes: in fact in our cases, MYC_target_v1 HGS was commonly enriched both in WT and RMS, and Hypoxia both in RMS and HB. In particular, should we address MYC_target_v1 HGS using druggable targets upstream the MYC protein such as CDK7, CDK9 or druggable targets involved in the control of MYC stability such as USP7, AURKA and PLK1, we would have success since all these druggable genes are expressed in all the samples of all the RMS and WT cases investigated. On the contrary, should we target the MYC-MAX complex, we would have success in all the cases but in 2 RMS and in 1 WT in which MAX is not expressed in all the samples of the tumor mass. Targeting Hypoxia in the cases of HB and RMS investigated would be successful only addressing PRKDC, which resulted to be expressed in all samples, whereas for CHEK1 the expression was heterogeneous in both histotypes. This suggests the possibility of target-driven therapeutical strategies in common among different histotypes. The increasing recognition of potentially actionable genetic alterations in key signaling pathways in pediatric tumors^35^ reinforces the value of implementing biologically driven therapies. Recently, the National Cancer Institute-Children’s Oncology Group Pediatric Molecular Analysis for Therapy Choice trial has proven the feasibility of a nationwide screening protocol for the identification of actionable genetic alterations and related assignments of children and adolescents with recurrent or refractory tumors to trials of molecularly targeted therapies^36^. For some tumors, many of the most frequent (epi)genetic events remain therapeutic challenges^36^.

As previously underlined, these clinical approaches always have to carefully consider the eventuality of the heterogeneous expression of the druggable target within the tumor mass. In addition to the ITH disclosed in the primary tumor, it is important to be aware of the differences that may occur between the primary tumor and the recurrence^12,13^: therapeutic decisions made on targets found investigating the primary tumor may not necessarily be rational when treating the recurrent disease.

Our study disclosed other commonalities among the investigated cases of 4 different histotypes: through WGCNA we identified a number of modules of genes sharing highly similar expression patterns across these different histotypes and across all the cases. These involve mRNA metabolic and catabolic processes, ribonucleoprotein complex, ribosome, translational processes, protein targeting to membrane/endoplasmic reticulum, cell cycle and mismatch repair, immune system, mRNA splicing and processing, intracellular transport and cellular localization.

We acknowledge the limits of this study, which is based on few cases for each of the investigated histotypes, and in which also the number of FFPE blocks sampled and investigated cannot be considered representative of the entire tumor bulk. We also recognize the very preliminary and explorative nature of our data, however, we are confident that some suggestive conclusions may be drawn. The ITH highlighted with our work has been demonstrated in all the four different histotypes and in all the cases investigated. In addition, we disclosed that, although different and distinct, diverse histotypes might share common elements, such as particular HGS and modules. This leads to the possibility to treat different clinicopathological entities with the same targeted drugs addressing these commonalities, leading to a shift from a disease-based to a target-based treatment, in an agnostic approach. Further investigations on larger number of cases and samples are needed to disclose which these common elements might be.

We believe that a warning that derives from our study is that to be aware of the ITH is mandatory and this ITH necessary impacts the real efficacy of any targeted therapy on the entire tumor mass.

## Supplementary Information

Supplementary Information 1.

Supplementary Information 2.

Supplementary Information 3.

Supplementary Information 4.

## Data Availability

All microarray data were compliant to MIAME (Minimum Information About a Microarray Experiment) guidelines and were deposited into the Gene Expression Omnibus (GEO) database of NCBI (National Center for Biotechnology Expression) (http://www.ncbi.nlm.nih.gov/geo/), with accession numbers GSE197147.
