# Supplementary material for: Gene expression-based dissection of inter-histotypes, intra-histotype and intra-tumor heterogeneity in pediatric tumors
Source: Sci Rep. 2022 Oct 25;12:17837. doi: 10.1038/s41598-022-20536-6 (PMC9596396; doi:10.1038/s41598-022-20536-6)
Supplement: Supplementary file 1 — Supplementary Information 1. [file 41598_2022_20536_MOESM1_ESM.pdf]

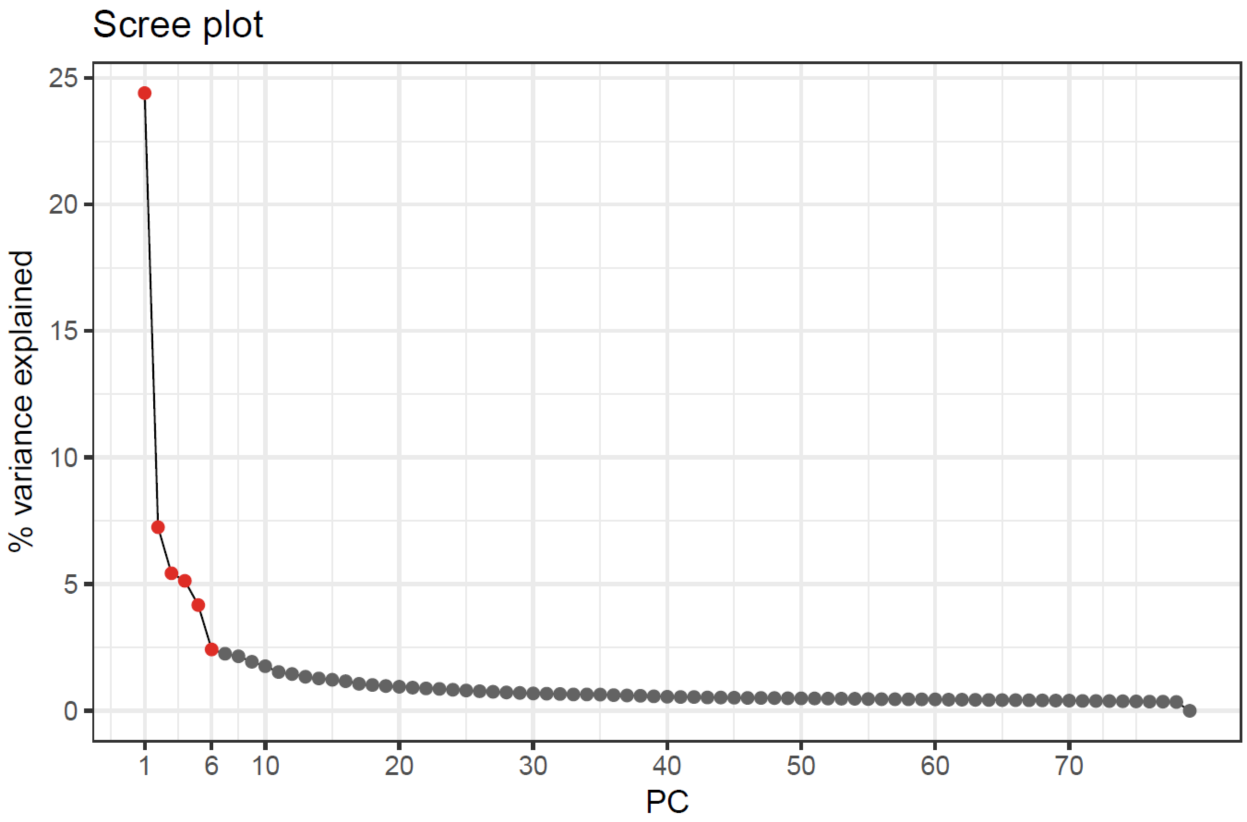

Supplementary Figure 1: A scree plot representing principal components (PC, x-axis) and the percentage of variance explained by each one (y-axis).

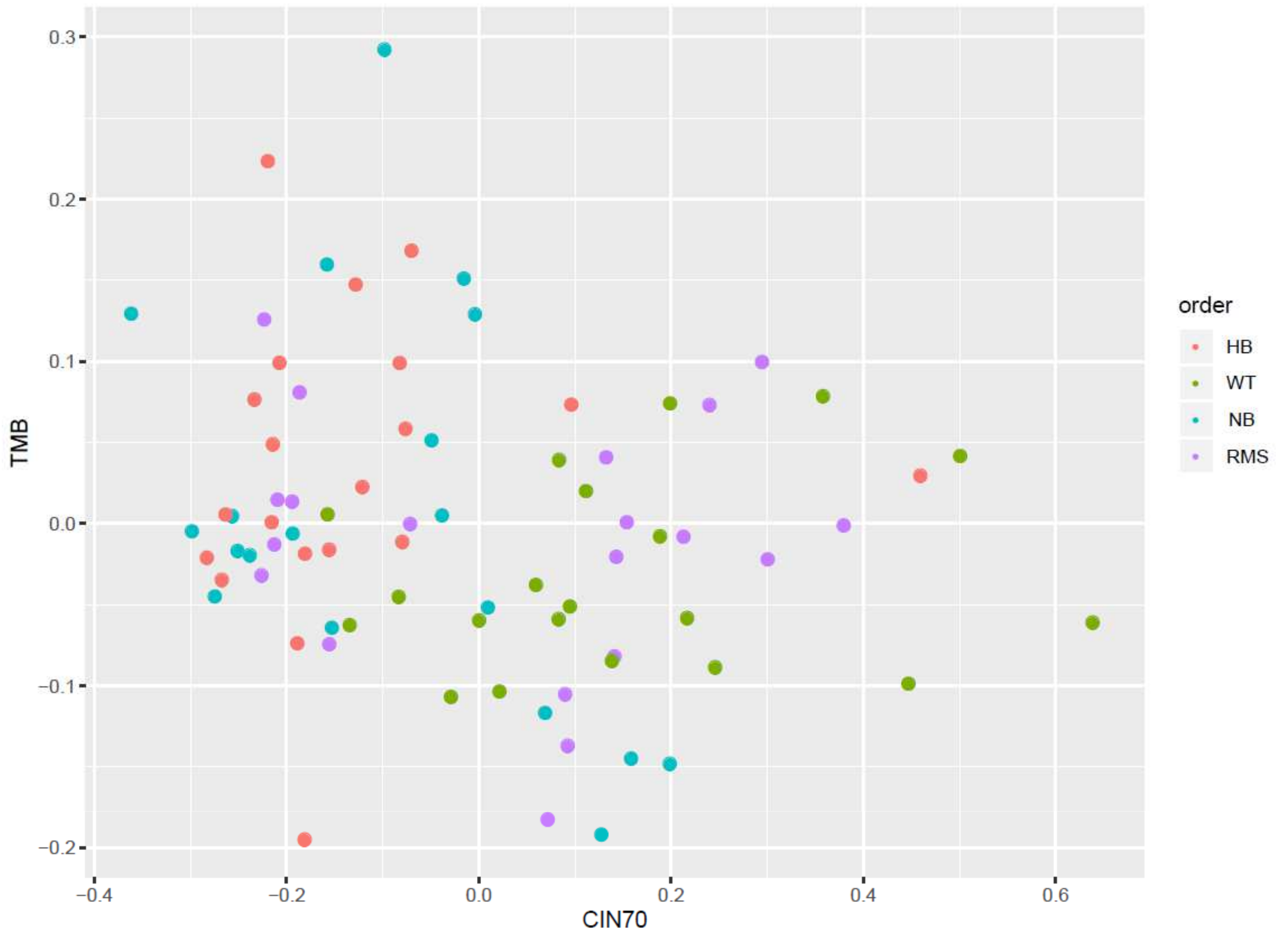

Supplementary Figure 2: Scatter plot of tumor mutation burden (TMB) and CIN70 scores.

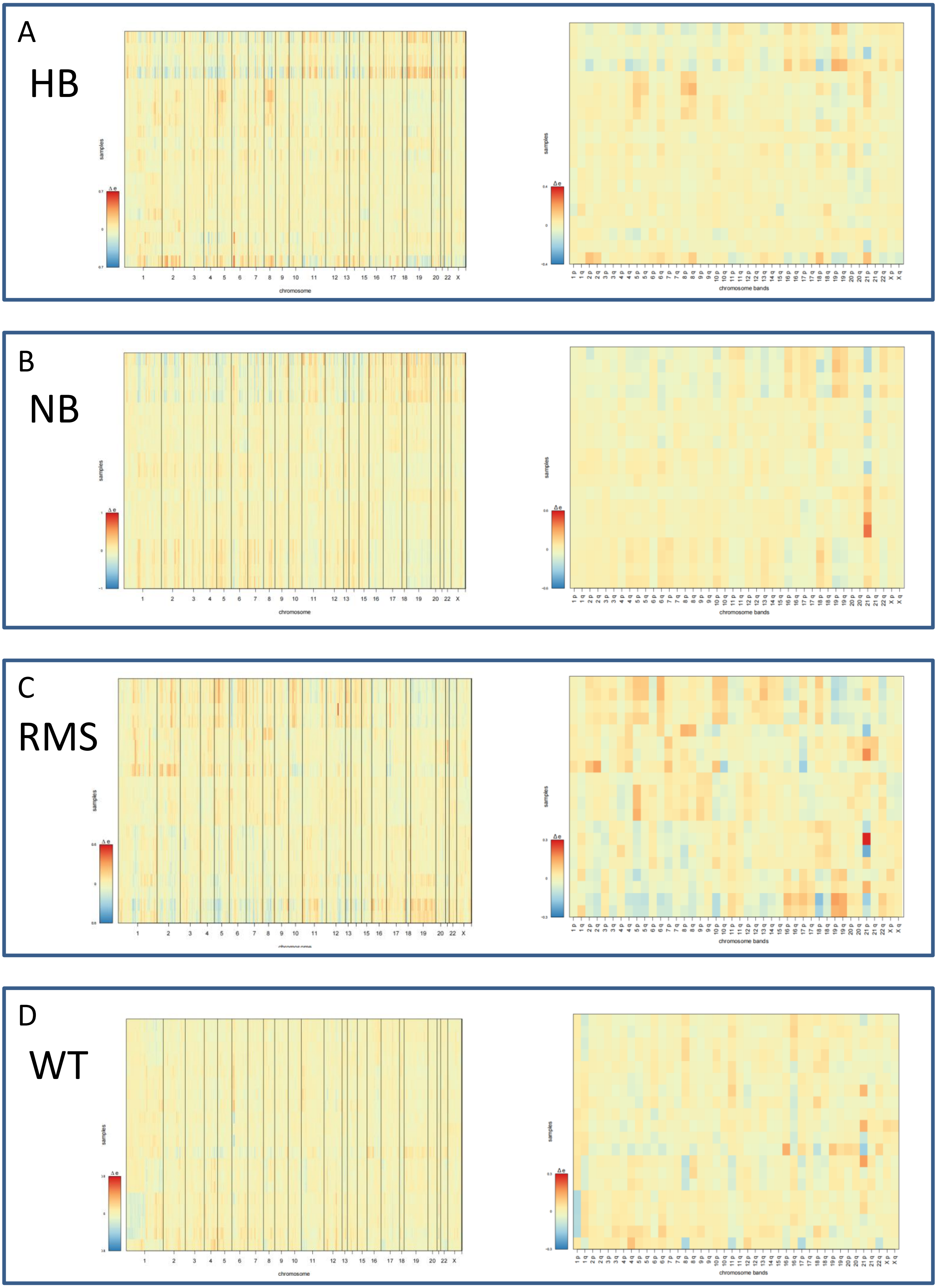

Supplementary Figure 3. Copy number alterations.

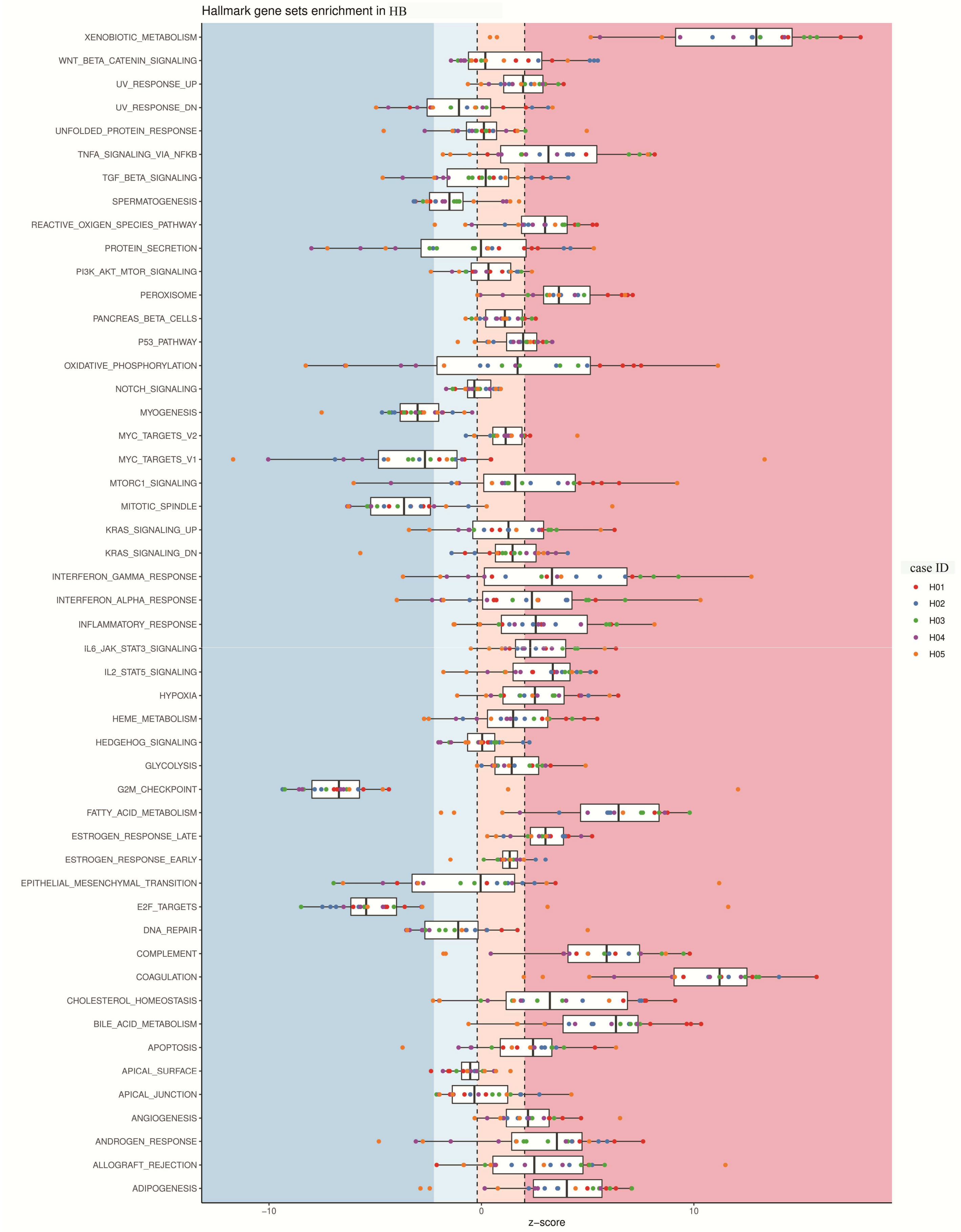

Supplementary Figure 4: Single Sample Hallmark Gene Sets (HGS) Analysis in hepatoblastoma.

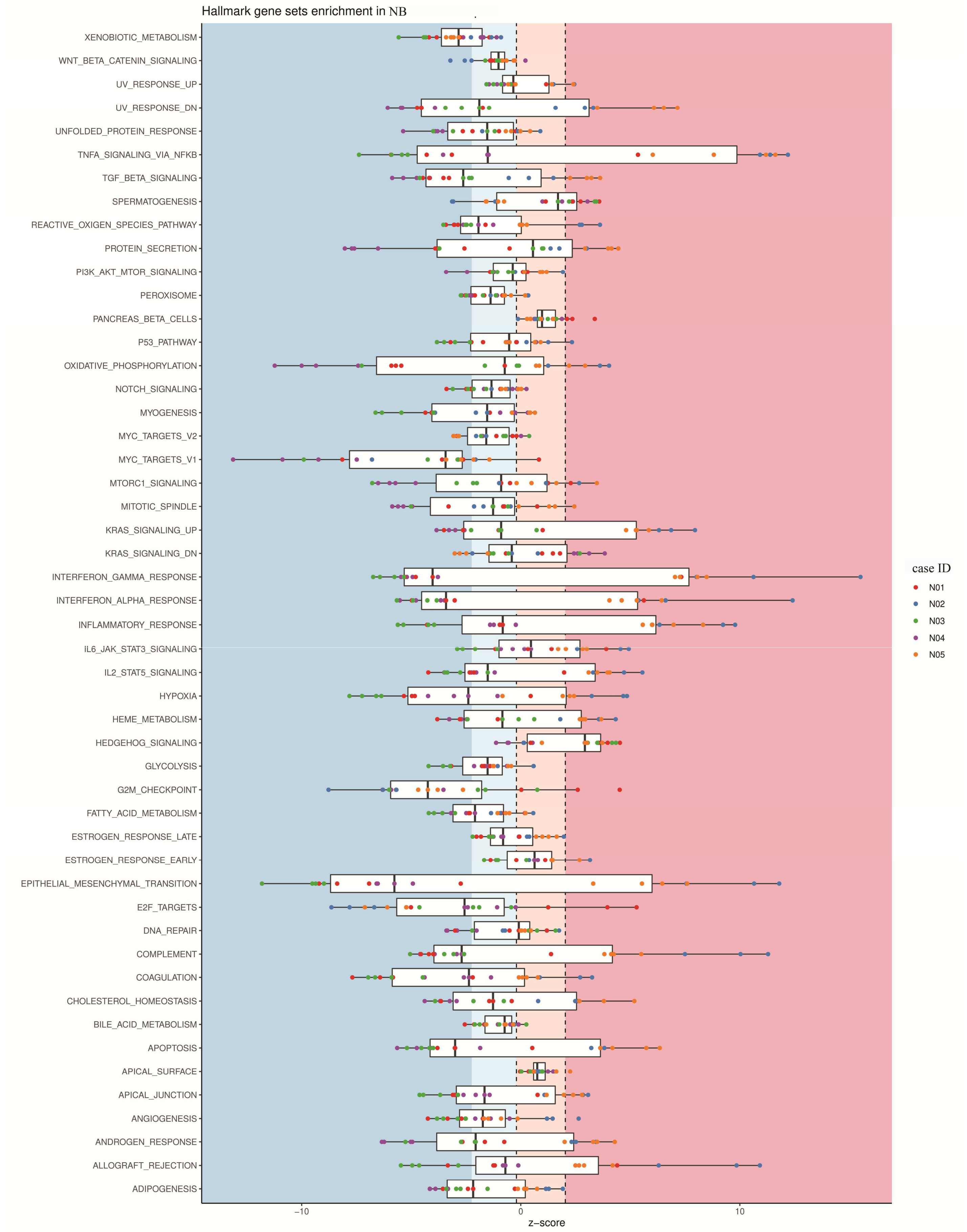

Supplementary Figure 5: Single Sample Hallmark Gene Sets (HGS) Analysis in neuroblastoma.

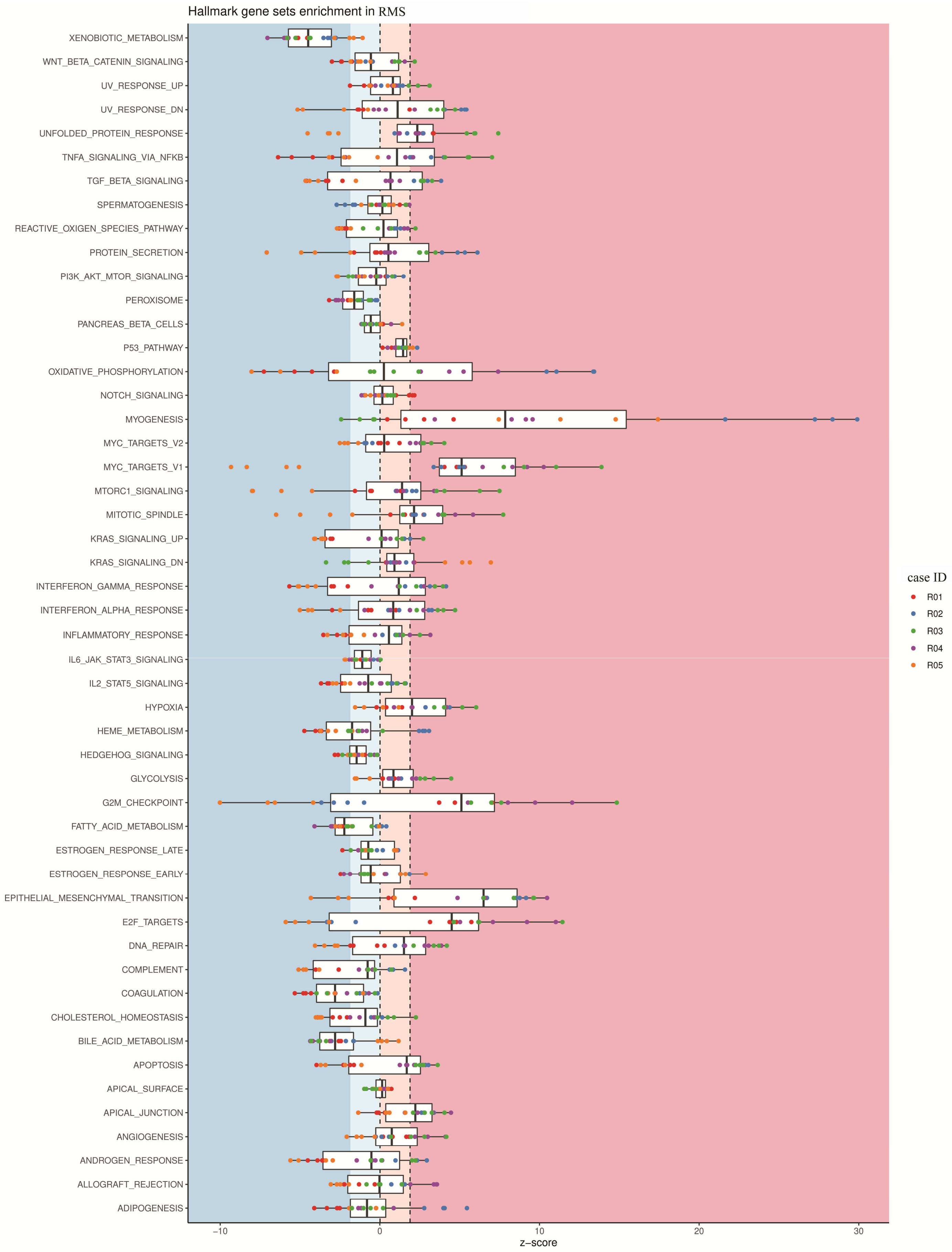

Supplementary Figure 6: Single Sample Hallmark Gene Sets (HGS) Analysis in rhabdomyosarcoma.

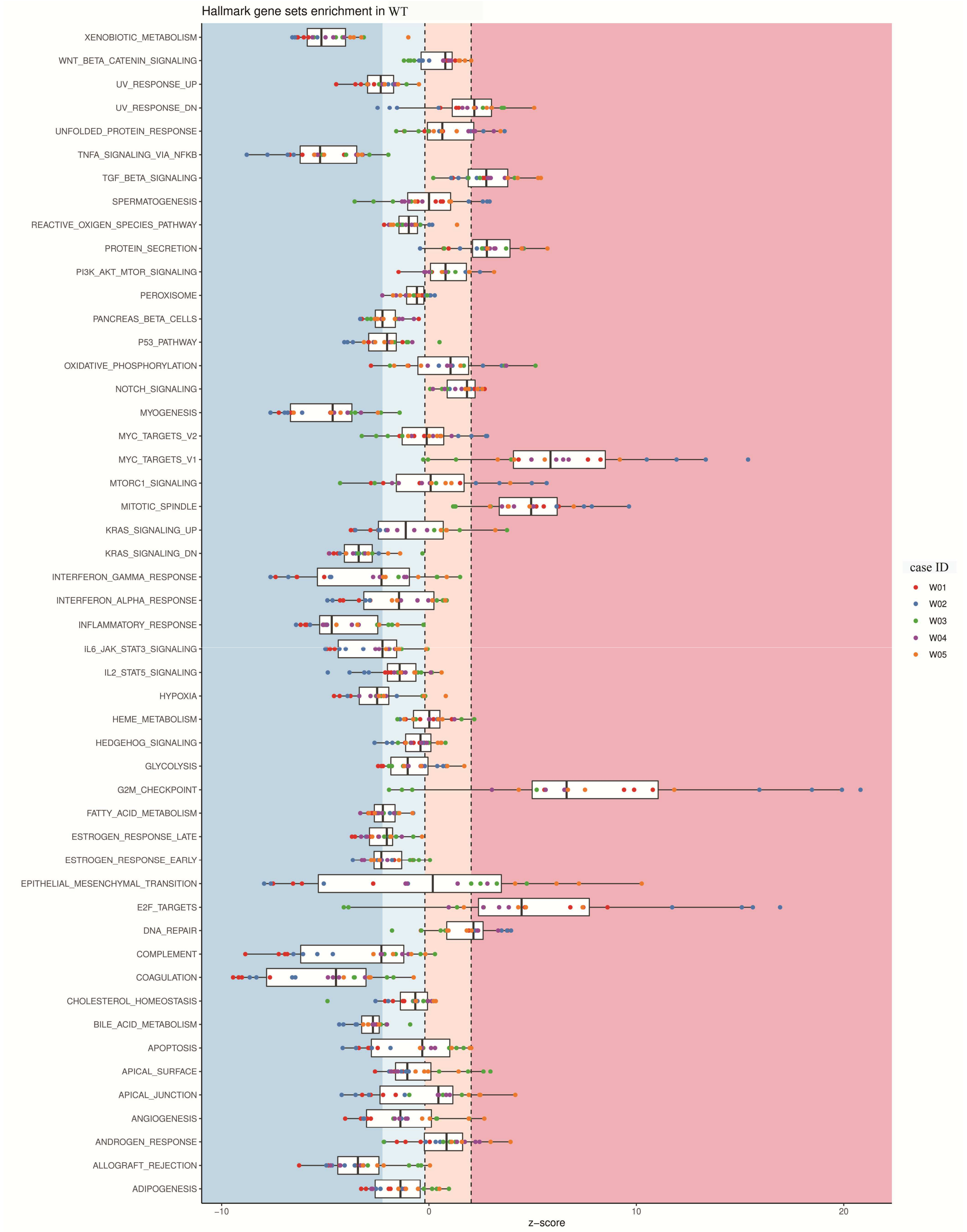

Supplementary Figure 7: Single Sample Hallmark Gene Sets (HGS) Analysis in Wilms tumor.

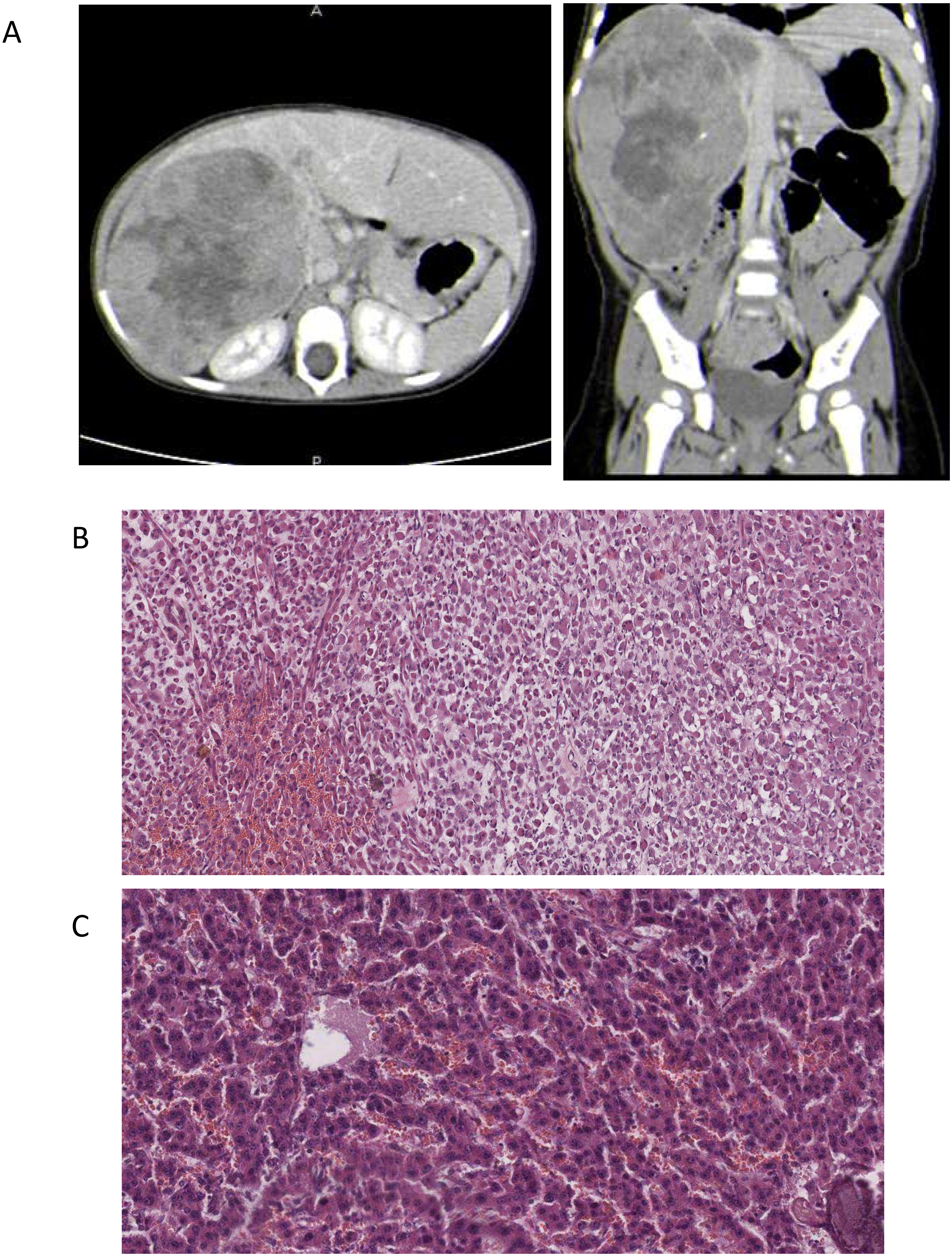

Supplementary Figure 8: Radiological and histological picture of case H01

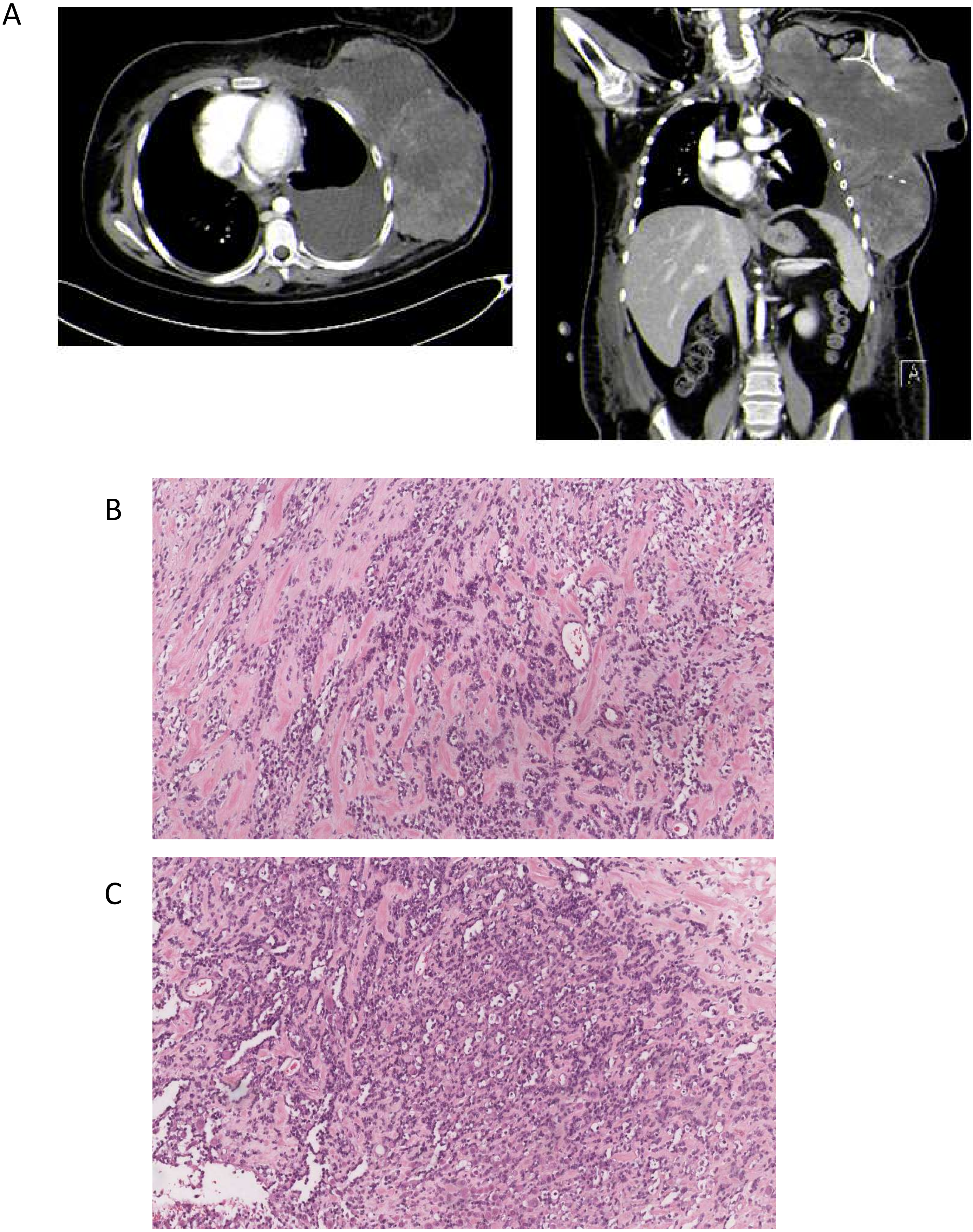

Supplementary Figure 9: Radiological and histological picture of case R01

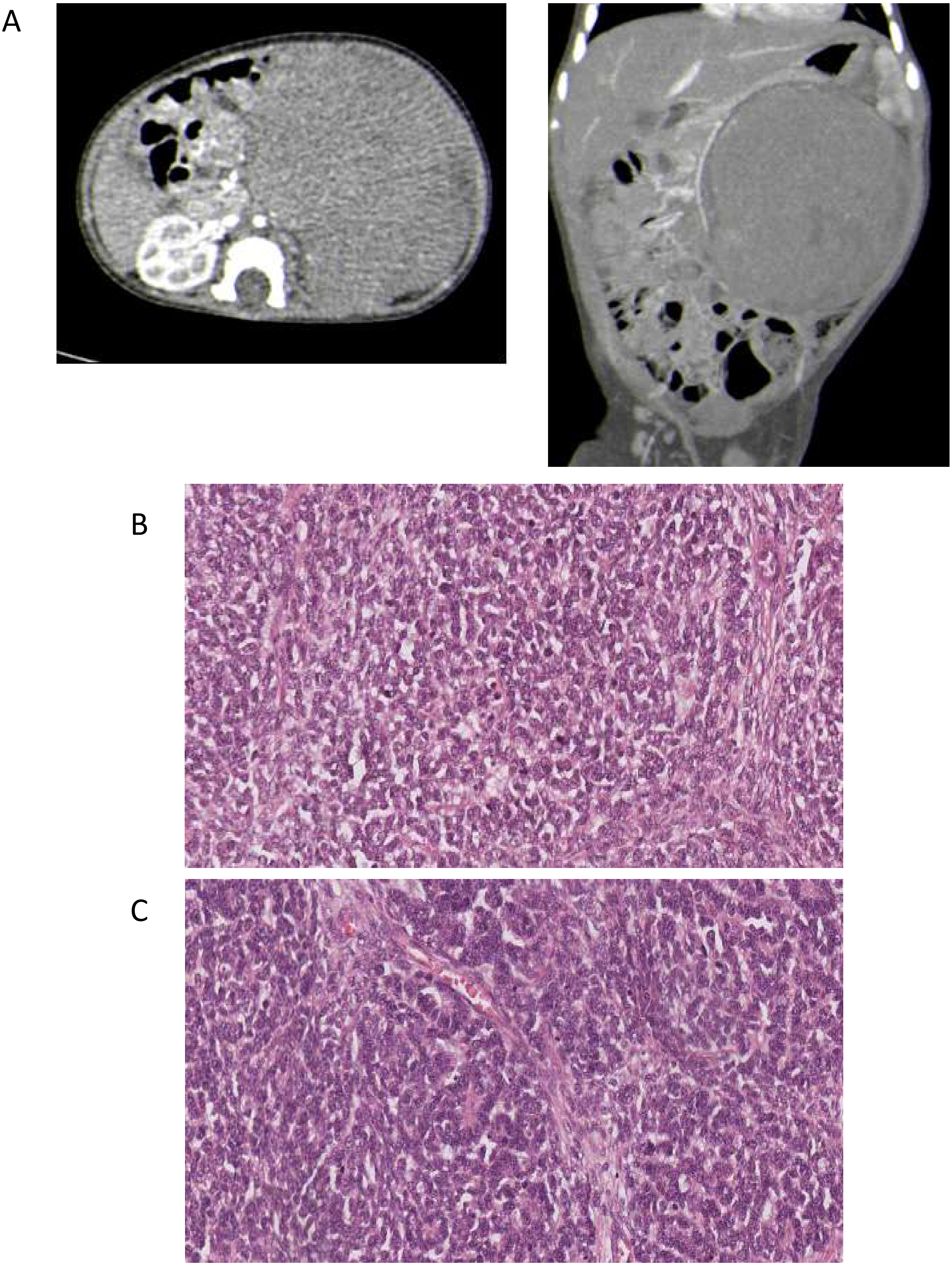

Supplementary Figure 10: Radiological and histological picture of case W01

Supplementary Figure legends

Supplementary Figure 1: Scree Plot. A scree plot representing principal components (PC, x-axis) and the percentage of variance explained by each one (y-axis).

Supplementary Figure 2: Scatter plot of tumor mutation burden (TMB) and CIN70 scores.

Hepatoblastoma (HB): CIN70, mean -0.13 (range -0.28, +0.46); TMB, mean 0.035 (range -0.19, +0.22)

Neuroblastoma (NB): CIN70, mean -0.093 (range -0.36, +0.20); TMB, mean 0.0072 (range -0.19, + 0.29)

Rhabdomyosarcoma (RMS): CIN70, mean 0.04 (range -0.22, +0.38); TMB, mean 0.01 (range - 0.18, +0.13)

Wilms tumor (WT): CIN70, mean 0.15 (range -0.15, +0.64); TMB, mean 0.032 (range -0.11, +0.08)

Supplementary Figure 3: Copy number alterations.

The plots show specific over- and under-enrichment at chromosome intervals or spanning p and q chromosome arms. Δe is the differential enrichment with respect to the mean expression of each gene averaged over all samples.

A) Hepatoblastoma (HB); Chromosome band spanning: $-0.7<\Delta e<+0.7$, Chromosome arm: $-0.4<\Delta e<+0.4$

B) Neuroblastoma (NB); Chromosome band spanning: $-1<\Delta e<+1$, Chromosome arm: $-0.7<\Delta e<+0.6$

C) Rhabdomyosarcoma (RMS); Chromosome band spanning: $-0.6<\Delta e<+0.6$, Chromosome arm: $-0.3<\Delta e<+0.3$

D) Wilms tumor (WT); Chromosome band spanning: $-0.8<\Delta e<+0.8$, Chromosome arm: $-0.3<\Delta e<+0.3$

Supplementary Figure 4: Single Sample Hallmark Gene Sets (HGS) Analysis in hepatoblastoma. Boxplots representing the enrichment of the 50 HGS (y-axis) through single sample z-scores (x-axis). Samples (dots) are colored by case ID. The four regions with color ranging from light blue to light red are bounded by the z-scores quartiles.

Supplementary Figure 5: Single Sample Hallmark Gene Sets (HGS) Analysis in neuroblastoma. Boxplots representing the enrichment of the 50 HGS (y-axis) through single sample z-scores (x-axis). Samples (dots) are colored by case ID. The four regions with color ranging from light blue to light red are bounded by the z-scores quartiles.

Supplementary Figure 6: Single Sample Hallmark Gene Sets (HGS) Analysis in rhabdomyosarcoma. Boxplots representing the enrichment of the 50 HGS (y-axis) through single sample z-scores (x-axis). Samples (dots) are colored by case ID. The four regions with color ranging from light blue to light red are bounded by the z-scores quartiles.

Supplementary Figure 7: Single Sample Hallmark Gene Sets (HGS) Analysis in Wilms tumor. Boxplots representing the enrichment of the 50 HGS (y-axis) through single sample z-scores (x-axis). Samples (dots) are colored by case ID. The four regions with color ranging from light blue to light red are bounded by the z-scores quartiles.

Supplementary Figure 8: Radiological and histological picture of case H01

A: lesion characterized by disomogeneus enhancement with necrotic component; B: rhabdomyoblastic component of hepatoblastoma; C: epithelial component of hepatoblastoma

Supplementary Figure 9: Radiological and histological picture of case R01

A: lesion characterized by disomogeneus enhancement with large necrotic component; B, C: presence of similar neoplastic cells in sclerohyaline stroma thrughout the tumor mass

Supplementary Figure 10: Radiological and histological picture of case W01

A: lesion characterized by homogeneus enhancement with small necrotic component; B, C: presence of admixture of blastemal, epithelial and stromal components throughout the tumor mass

Supplementary Tables

Supplementary table 1: Clinical, radiological and pathological data of the selected patients, and microscopical description of the samples.

histotype	patient ID	tumor site	classification	stage	radiology*	sample ID	components in each FFPE block
HB	H01†	liver	pretreated HB	IV	lesion with moderate inhomogeneous contrast enhancement due to necrotic component (CT examination)	KF42	epithelial, mesenchymal; rhabdomyoblasts
						KF43	epithelial, mesenchymal
						KF44	epithelial, mesenchymal
						KF45	epithelial, mesenchymal
	H02	liver	pretreated HB	standard risk	lesion with high homogeneous contrast enhancement (CT examination)	KF46	epithelial
						KF47	epithelial
						KF48	epithelial
						KF49	epithelial
	H03	liver	pretreated HB	standard risk	lesion with high homogeneous contrast enhancement (CT examinatio	KF50	epithelial
						KF51	epithelial
						KF52	epithelial
						KF53	epithelial
	H04	liver	pretreated HB	standard risk	lesion with moderate inhomogeneous contrast enhancement (CT examination)	KF54	epithelial
						KF55	epithelial, mesenchymal
						KF56	epithelial
						KF57	epithelial
	H05	liver	pretreated HB	standard risk siopel	lesion with moderate inhomogeneous contrast enhancement (CT examination)	KF58	epithelial, mesenchymal; HCC-like foci
						KF59	epithelial, mesenchymal
						KF60	epithelial
						KF61	epithelial, mesenchymal; chondroblasts
NB	N01	left adrenal gland	untreated poorly differentiated NBL	L1	lesion with homogeneous moderate contrast ehancement (CT examination)	KF22	poorly differentiated NBL
						KF23	differentiating NBL and immature ganglioneuroma
						KF24	poorly differentiated and differentiating NBL
						KF25	poorly differentiated NBL
	N02†	right adrenal gland	pretreated NBL	M	lesion with homogeneous low contrast ehancement (CT examination)	KF26	differentiating NBL
						KF27	undifferentiated and differentiating NBL
						KF28	differentiating NBL
						KF29	
	N03	left adrenal gland	untreated undifferentiated NBL with pleomorphic cells	L1	lesion with homogeneous moderate contrast enhancement (MR examination)	KF30	undifferentiated NBL; pleomorphic cells
						KF31	undifferentiated NBL
						KF32	undifferentiated NBL; pleomorphic cells
						KF33	undifferentiated NBL; pleomorphic cells
	N04	right adrenal gland	untreated undifferentiated NBL	L2	lesion with homogeneous moderate contrast enhancement (MR examination)	KF34	undifferentiated NBL
						KF35	undifferentiated NBL
						KF36	undifferentiated NBL
						KF37	undifferentiated NBL
	N05	right adrenal gland	pretreated NBL	M	lesion with homogeneous moderate contrast enhancement (MR examination)	KF38	ganglioneuroblastoma intermixed
						KF39	ganglioneuroblastoma intermixed
						KF40	ganglioneuroblastoma intermixed
						KF41	ganglioneuroblastoma intermixed

RMS	R01†	periscapular region soft tissue	sclerosing RMS	III	lesion with moderate inhomogeneous contrast enhancement due to necrotic component (CT examination)	KE82	classic; rhabdomyoblastic differentiation
						KE83	classic; rhabdomyoblastic differentiation
						KE84	classic; rhabdomyoblastic differentiation
						KE85	classic; rhabdomyoblastic differentiation
	R02	forearm soft tissue	ARMS	IV	lesion with moderate inhomogeneous contrast enhancement (CT examination)	KE86	classic; rhabdomyoblastic differentiation
						KE87	classic; rhabdomyoblastic differentiation
						KE88	classic; rhabdomyoblastic differentiation
						KE89	classic; rhabdomyoblastic differentiation
	R03	prostatic region soft tissue	ERMS with diffuse anaplasia	III	lesion with moderate inhomogeneous contrast enhancement (MR examination)	KE90	classic; rhabdomyoblastic differentiation; anaplasia
						KE91	classic; rhabdomyoblastic differentiation; anaplasia
						KE92	classic; rhabdomyoblastic differentiation
						KE93	classic; rhabdomyoblastic differentiation; anaplasia
	R04†	right calf soft tissue	pretreated ARMS	IV	lesion with moderate inhomogeneous contrast enhancement due To diffuse necrosis (MR examination)	KE94	classic morphology
						KE95	classic morphology
						KE96	classic morphology
						KE97	classic morphology
	R05	left thight soft tissue	ARMS	III	lesion with moderate inhomogeneous contrast enhancement due To diffuse necrosis (MR examination)	KE98	classic; rhabdomyoblastic differentiation
						KE99	classic; rhabdomyoblastic differentiation
						KF00	classic; rhabdomyoblastic differentiation
						KF01	classic; rhabdomyoblastic differentiation
WT	W01	left kidney	pretreated WT without anaplasia (B,E,S)	II	lesion with moderate homogeneous contrast enhancement with minimal necrosis (CT examination)	KF02	blastemal, epithelial, stromal
						KF03	blastemal, epithelial, stromal
						KF04	blastemal, epithelial, stromal
						KF05	blastemal, epithelial, stromal
	W02	left kidney	pretreated WT without anaplasia (E,S,B)	IV	lesion with high inhomogeneous contrast enhancement(CT examination)	KF06	epithelial, stromal, blastemal
						KF07	epithelial, stromal, blastemal
						KF08	epithelial, stromal, blastemal
						KF09	epithelial, stromal, blastemal
	W03	left kidney	pretreated WT without anaplasia (E,S,B)	I	lesion with high inhomogeneous contrast enhancement due to diffuse necrosis(CT examination)	KF10	stromal, epithelial, blastemal
						KF11	stromal, epithelial, blastemal
						KF12	stromal, epithelial, blastemal
						KF13	stromal, epithelial,blastemal, adipous tissue
	W04	left kidney	pretreated WT without anaplasia (S)	II	lesion with homogeneous low contrast enhancement (CT examination)	KF14	stromal
						KF15	stromal
						KF16	stromal
						KF17	stromal, epithelial
	W05	right kidney	untreated WT with diffuse anaplasia (S)	II, High Risk for diffuse anaplasia	lesion with homogeneous low contrast ehancement (CT examination)	KF18	stromal, diffuse anaplasia
						KF19	stromal, diffuse anaplasia
						KF20	stromal, diffuse anaplasia
						KF21	stromal, diffuse anaplasia

HB: hepatoblastoma; NB: neuroblastoma; RMS: rhabdomyosarcoma (ARMS: alveolar RMS; ERMS: embrional RMS); WT: Wilms Tumor; † expired; B: blastemal; E: epithelial; S: stromal; CT: computed tomography; MR: magnetic resonance.

* Tumor vascularity is correlated radiologically to contrast enhancement: some tumors as WT or HB are generally characterized by high contrast enhancement that can be homogeneous or inhomogeneous (also for the presence of necrosis) but some times WT or HB can be also characterized by moderate contrast enhancement due to minor vascularity. NB and RMS are generally characterized by low-moderate contrast enhancement and in particular in RMS the enhancement is inhomogenous also for the presence of necrosis.

Supplementary Table 2: distance metrics.

Parameter	Description	Distance Metric
Mean pairwise distance	mean distance between two data points belonging to the same histotype or tumor	$mean_{i,j}\ d_{ij}$
Max pairwise distance	maximum distance between data points belonging to the same histotype or tumor	$max_{i,j}\ d_{ij}$
Mean centroid distance	mean distance between a data point and its centroid	$mean_{i}\ d_{ic}$
Max centroid distance	maximun distance between a data point and its centroid	$max_{i}\ d_{ic}$

Supplementary Table 3: Intra-histotype and intra case distances

histotype	case	max centroid distance (case)	max centroid distance (histotype)	mean centroid distance (case)	mean centroid distance (histotypes)	max pairwise distance (case)	max pairwise distance (histotype)	mean pairwise distance (case)	mean pairwise distance (histotype)
	H01	47,522		37,059		40,442		26,699	
	H02	55,969		43,335		79,85		47,133	
HB	H03	31,528	163,66	24,551	53,393	33,522	277,58	25,811	79,399
	H04	119,83		68,994		128,21		77,958	
	H05	163,66		93,027		277,58		156,02	
	N01	68,368		58,099		49,477		37,215	
	N02	82,585		66,413		59,634		40,844	
NB	N03	72,669	286,08	63,431	93,993	84,666	364,01	46,685	133,16
	N04	286,08		177,96		241,14		154,53	
	N05	105,11		97,169		31,145		22,435	
	R01	58,659		54,349		25,129		18,096	
	R02	88,266		85,206		32,431		20,122	
RMS	R03	59,451	115,3	32,458	55,667	53,286	193,65	30,663	80,217
	R04	32,441		26,059		22,657		16,289	
	R05	115,3		80,261		85,176		59,401	
	W01	34,595		26,534		50,063		29,773	
	W02	53,763		44,269		50,718		33,406	
WT	W03	66,926	76,731	39,371	38,854	71,464	135,3	39,347	55,179
	W04	76,731		48,692		51,597		28,96	
	W05	42,88		35,406		68,514		39,928	

Supplementary Table 2: distance metrics. d_{ij} = distance between i and j; d_{ic} = distance between i and its centroid

Supplementary Table 3: Intra-histotype and intra-case distances. Four types of heterogeneity statistics are reported: (i) max centroid distance, (ii) mean centroid distance (iii) max pairwise distance (iv) mean pairwise distance. Euclidean distances were computed by considering the first six principal components, which together explain about 49% of the total variance. The four summary statistics are computed both among samples from the same case (ITH) and among samples from the same histotype (intra-histotype).

Supplementary Table 4: Single sample HGS expression in the four investigated histotypes.

Supplementary Table 5: Expression levels of selected genes belonging to druggable pathways commonly expressed in each histotype (at least 16/19-20 blocks). Patients and samples identifiers are indicated.

Supplementary Table 6: Enriched Pathways in Gene Modules.
